# Compensation

**Published:** 1871-02

**Authors:** 


### HYGENIC AND MISCELLANEOUS INTELLIGENCE.

### COMPENSATION.

Compensation, in the workings of organic law as to the human body, is strikingly illustrated by George Combe, in the eighth chapter of his admirable book on "The Constitution of Man." He shows that the penalties for violated law often most clearly exhibits the design of the law itself. The organic law of *Vital Sensation* is under consideration. We give the general thought and not the language: In the long time ago, when Jupiter, *pater*, ruled the world, and fables were facts, a poor plowman lay on his couch racked with pain and burning with fever. He cried out in his anguish: "O Jupiter, O Jupiter!" The god came at his cry. "What wouldst thou, my child?" was the kind inquiry. "Thou art unreasonable and cruel in thy laws, O Jupiter! I was plowing in my field, which thy laws make needful, in order to support myself and my family; and while thus employed, a storm of thy rain drenched me and a cold blast of thy wind chilled me, and now I lie agonized with excruciating pain. Compassionately Jupiter replied: "I see, my poor child; thou dost complain of my law of sensation, common to thee and all my sentient creatures. Shall I release thee from the operations of this law, so far as thyself art concerned, both as to its penalties and its rewards as well? At thy wish and on these conditions, shalt thou be quite relieved from pain." "Most kind and mighty Jupiter," earnestly exclaimed the sufferer, "for such a boon thou wilt have my highest and most lasting gratitude!" "Be it so," replied the god. The plowman was at once restored to perfect health, and again follows his team a-field. How great must now be his happiness! The law of sensation is no longer applicable to him. Fever will no more burn up his blood; pain in none of its sharp agonies will ever again course through any part of his body. How little did he understand that in thus curing pain he also made all pleasure impossible. As before, the blood poured its vital currents through his veins, the air came to his lungs, light to his eyes, sounds to his ears, but he heeded them not. The law of sensation was suspended as to him. He went to his home; no sweet voice of his wife or child greeted his coming. The loving kiss and fond embrace gave back no emotion, Tenderness and affection no more swelled his bosom. What consternation and distress unutterable now fills his soul. "O Jupiter, O Jupiter!" was again the intensified outburst of his wretched heart. Again the god listened to his call. "What now, my miserable child? Art thou still not content, since at thy wish I even set aside my law of sensation so far as applied to thee? What wouldst thou now?" The poor man recited his sad experience while exempt from the law of sensation, and cried out: "Anything rather than this." "Wouldst thou then be returned to thy couch of pain, to accept and bear patiently the penalty of my violated law? Wilt thou also more wisely and carefully seek to obey all my laws?" "Most gratefully, O Jupiter, do I accept your clemency and promise obedience."

The same principle of compensation is equally true of every every organic law not only, but all laws of the blessed and infinite Law-maker.—*Herald of Health.*

